# Chemosystematic Value of the Essential Oil Composition of *Thuja* species Cultivated in Poland—Antimicrobial Activity

**DOI:** 10.3390/molecules14114707

**Published:** 2009-11-19

**Authors:** Dimitroula Tsiri, Konstantia Graikou, Loretta Pobłocka-Olech, Miroslawa Krauze-Baranowska, Caroline Spyropoulos, Ioanna Chinou

**Affiliations:** 1 Division of Pharmacognosy and Chemistry of Natural Products, School of Pharmacy, University of Athens, Athens 15771, Greece; E-Mails: dtsiri@yahoo.com (D.T.); kgraikou@pharm.uoa.gr (K.G.); ichinou@pharm.uoa.gr (I.C.); 2 Department of Botany, Faculty of Biology, University of Athens, Athens 15771, Greece; E-Mail: cgspyro@biol.uoa.gr (C.S.); 3 Department of Pharmacognosy, Medical University of Gdańsk, Gen. J. Hallera 107 str., 80-416 Gdańsk, Poland; E-Mails: loretta@amg.gda.pl (P-O.L.); krauze@amg.gda.pl (K-B.M.)

**Keywords:** *Thuja occidentalis*, *Thuja plicata*, chemosystematics, volatiles, antimicrobial activity

## Abstract

In the framework of the correlation between chemotaxonomy and chemical analysis studies, the chemical composition of the essential oils of four varieties of *Thuja* species cultivated in Poland − *T. occidentalis ‘globosa’*, *T. occidentalis ‘aurea’*, *T. plicata* and *T. plicata* *‘gracialis’* − were investigated by GC and GC-MS. Thirty-one compounds were identified from *T. occidentalis ‘globosa’*, representing 96.92% of the total oil; twenty-seven from *T. occidentalis ‘aurea’* (94.34%); thirty-one from *T. plicata* (94.75%); and thirty compounds from *T. plicata* *‘gracialis’* (96.36%). The main constituents in all samples were the monoterpene ketones *α*- and *β*-thujone, fenchone and sabinene, as well as the diterpenes beyerene and rimuene. The chemosystematic value of the total ketone content of all samples (which varied from 54.30–69.18%) has been discussed and investigated. The constituents, beyerene and the mixture of *α*- and *β*-thujone, were isolated from the oils and tested against six Gram-positive and -negative bacteria and three pathogenic fungi. The oils of the two *T. plicata* species exhibited significant antimicrobial activity, while the mixture of *α*- and *β*-thujone showed very strong activity as well.

## Introduction

*Thuja* is a small genus of the Cupressaceae family comprising five extant species, of which *T. occidentalis* L. and *T. plicata* Donn ex D. Don are widely grown in western and eastern North America and Europe [1]. *T. occidentalis* (Eastern arborvitae, Atlantic red cedar) which comprises shrubs or trees up to 15–35 meters tall, with spreading crowded branches, occurs in eastern North America, occupying a geographical range between the sub Arctic taiga-tundra interface in the north and the belt of deciduous angiosperm forests in the south, while in Europe it is grown as an ornamental tree [2]. Leaves are evergreen, scale-like, bright green above and pale green below. *T. plicata* (Western arbor-vitae, Pacific red cedar), a tree 50–70 meters tall with arching branches and leaves that are glossy green above and white-striped on the lower surface, is distributed in the Pacific Coastal Mountains and the Rocky Mountains of western North America [1]. Both species are used as a commercial crop and are managed for timber in Europe and North America. In folk medicine, *Thuja occidentalis* has been used to treat bronchial catarrh, enuresis, cystitis, psoriasis, uterine carcinomas, amenorrhea and rheumatism [1]. Cedar leaf oil can be obtained by steam distillation or hydrodistillation of the foliage and is used for the production of perfumes, insecticides, soaps and deodorants [3,4] The major constituent of the oil, the monoterpene thujone, is used pharmacologically as an active ingredient in the production of nasal decongestants and cough suppressants, perfumes, shoe polishes and soaps, while many cultivars are grown for ornamental purposes [5].

The oil of western red cedar leaves (*T. plicata*) was first investigated by Brandel, who reported the presence of thujone, fenchone, and esters of borneol [6]. In 1912, Rose and Livingston confirmed the major constituent to be *Z*-thujone (80–85%), and identified α-pinene, thujyl alcohol and thujyl acetate but could not isolate fenchone or bornyl esters [6]. Guenther points out that thujone occurs in nature as a mixture of the stereoisomers *Z*-thujone and *E*-isothujone, which has been confirmed by Rudloff [6] for *T. plicata* (*Z*-thujone 77.5% and *E*-isothujone 7.8%) and later [7] for 29 populations from the USA. 

The oil of eastern cedar leaves (*T .occidentalis)* has been independently investigated by Shaw [6] and Rudloff [6], who reported the thujone fraction as a mixture of *Z*-thujone and *E*-isothujone, while Keita *et al*. [8] in their analyses, reported twenty-two compounds including *α*-thujone (= *Z*-thujone) (49.64%), fenchone (14.06%) and *β*-thujone (= *E*-isothujone) (8.98%) as the most abundant compounds.

Regarding *Thuja* species growing in Europe, the essential oils from the wood from various *Thuja* species occurring in Czechoslovakia have been studied before [9], as well as the leaf oil from two different harvests of Slovakian *T. occidentalis* L. ‘*malonyana’* (Table 2) [10]. The aim of this study was the investigation of the chemical composition (Table 1) of the essential oil of the leaves of two *Thuja* species cultivated in Poland (*T. occidentalis* and *T. plicata*). The four different varieties are *T. occidentalis* “aurea”, *T. occidentalis* “globosa”, *T. plicata* and *T. plicata* “gracialis”. We also report the isolation of their abundant terpenes (beyerene and *α*- and *β*-thujone) and the antimicrobial assay of the oils and the isolated terpenes (Table 3).

## Results and Discussion

The oils from dried *Thuja* leaves were light yellow, with distinct sharp odors and the yield varied from 0.58% v/w to 0.87% v/w. The main components of the essential oils of each variety of the two studied species with their percentages and their retention indices are listed in Table 1, while the results of the antibacterial and antifungal activities of the essential oils and their main components are presented in Table 3. Furthermore, a comparison of the previous reported data concerning the most abundant compounds in *T. occidentalis* and *T. plicata* leaf oils of different origin [2,6,7,8,10,11] with our data from Polish species is given in Table 2. 

The study of the four samples resulted in the identification of thirty-one compounds in the oil of *T. occidentalis* “globosa” (area 96.92%) while in the oil of and *T. occidentalis* “aurea” twenty-seven constituents have been identified (area 94.34%), having both as major constituents *α*-thujone (50.14 and 51.60%, respectively), beyerene (8.54% and 11.28%, respectively), sabinene (4.55% and 3.43% respectively) and camphor (4.47 and 3.09 % respectively). The characteristic difference between them is that *T. occidentalis* “globosa” has a high content of the ketones *β*-thujone and fenchone, while *T. occidentalis* “aurea” has high levels of the diterpene rimuene. Regarding the reported leaf oil of eastern white cedar (*T. occidentalis*) [11], the main terpene components are almost the same in comparison with our results, but the content of fenchone is much higher (12.80%). 

The chemical profiles of the oils of the two *T. plicata* were also comparable, as thirty-two compounds have been identified in *T. plicata* ( 94.75%) and thirty in the oil of *T. plicata* “gracialis” (96.36%) having also *α*-thujone (62.12% and 54.48%, respectively), *β*-thujone (7.06% and 6.39%), terpinen-4-ol (4.66% and 3.11%) and sabinene (6.00% and 2.94%) among the most abundant compounds. On the other hand, *T. plicata* shows higher content of the ketone fenchone, while *T. plicata* “gracialis” has high levels of the diterpene beyerene. According to our results, the analysis of the leaf essential oil of Canadian red cedar (*T. plicata*) [7], where thujone is the abundant compound (73.16%), but also the content of sabinene, terpinen-4-ol and isothujone was considerably high, was similar to this report. Notable is the absence of fenchone, which has a high percentage in our analysis (7.06%). 

The main monoterpenic ketones identified in all samples were *α*-thujone (50.14–62.12%), *β*-thujone (2.70–7.06%) and fenchone (0.17–7.06%). The total ketone content in the oil samples varied between 54.30% and 69.18%, while *T. plicata* and *T. plicata* “gracialis” showed the highest values (69.16% and 63.59% respectively), which meet the specifications of the Essential Oil Association (EOA): ketone content of the oil should be no less than 60% [5]. 

Leaf oil analysis for *Thuja* genus has been proved as a safe chemosystematic tool in the studies on different species and subspecies [12]. The chemosystematic value of the total ketone content, especially of thujone isomers and fenchone, is confirmed from this study, which is in accordance with the previous published data, for *Thuja* from different origin, as is shown in Table 2. It may, therefore be concluded that leaf oil analysis can be of considerable help, providing basic information needed for the chemosystematic approach of a genus.

### Antimicrobial activity

The reported essential oils and the isolated compounds have been assayed for their antimicrobial activity (Table 3). Previously, the insecticidal activity of the oil of *T. occidentalis* has been examined and it was characterized as highly toxic [13]. Also the antifungal activity, in order to control the biocontamination in libraries and archives storage areas, of thuja oil has been reported, showing that this oil had little inhibitory effect on the fungal combination [14].

In the antimicrobial screening, the oil of *T. plicata* exhibited the highest antibacterial (MIC values 0.50–1.25 mg/mL) as well as antifungal (MIC 0.87–1.12 mg/mL) activity, followed by that of *T. plicata* “gracialis” (MIC values 0.75–1.24 mg/mL and 1.15–1.45 mg/mL, respectively). This antimicrobial activity is suspected to be associated with the high percentage of *α*- and *β*-thujone, which exhibited also strong activities against the assayed microorganisms (MIC 0.09–0.83 mg/mL), and are well known as the main active compounds in many essential oils possessing similar antimicrobial properties [15,16]. The diterpene beyerene, has been isolated in this study, to our knowledge, for the first time from essential oil source, and showed interesting antimicrobial (MIC 0.87–1.37 mg/mL) and antifungal (MIC 1.15–1.50 mg/mL) activity as well. The results of this study, showing such an interesting antimicrobial profile, could support the potential use of plant essential oils, which could be of economical benefit.

## Conclusions

The chemosystematic value of the total ketone content, especially of thujone isomers and fenchone, is confirmed from this study, as oil analysis for *Thuja* genus has been proved as a safe chemosystematic tool in previous studies on different species and subspecies [12]. Therefore, it may be amplified that leaf oil analysis can be of considerable help, providing basic information needed for the chemosystematic approach of a genus.

The antimicrobial study of the essential oils and the isolated compounds showed an interesting antimicrobial profile, which could provide economical benefit with the potential use of plant essential oils.

## Experimental 

### General

GC Analysis: Analyses were carried out on a Perkin-Elmer Clarus 500 gas chromatograph with FID, fitted with a DB-5 capillary column (30 m × 0.25 mm.; film thickness 0.25 μm). The column temperature was programmed from 75 °C to 200 °C at a rate of 2.5 °C/min. The injector and detector temperatures were programmed at 230 °C and 300 °C, respectively.

GC/MS Analysis: The GC-MS analysis of the essential oils was performed using a Hewlett Packard 6890 series II gas chromatograph equipped with an HP-5 capillary column (30 m, 0.25 mm i.d., 0.25 μm film thickness) and a mass spectrometer 5973 of the same company which was operated on EI mode. Helium was the carrier gas at a flow rate of 1 mL/min. The injector was operated at 200 °C and the oven temperature was programmed as follows: 60 °C for 5 min, then gradually increased to 280 °C with a 3 °C/min rate. In all experiments the samples have been analysed at least twice and the results have been expressed as average. The identification of the compounds was based on comparison of their retention indices (RI), retention times (RT) and mass spectra with those from Wiley libraries spectra, the NIST/NBS and literature data [17,18,19]. 

### Plant material

The foliage samples of all *Thuja* species were collected from trees cultivated in the Medicinal Plants Garden of the Medical University in Gdańsk (Poland) in March 2003. Voucher specimens of the plants have been deposited in the Herbarium of the Medicinal Plants Garden of the Medical University of Gdańsk (Poland) with the following numbers : *Thuja occidentalis ‘globosa’* – 01-015, *T. occidentalis ‘aurea’* – 01-016, *T. plicata* – 01-017 and *T. plicata ‘gracialis’* – 01-018. The collected materials were air-dried at room temperature prior to hydrodistillation. 

### Oil isolation 

The plant material of each plant (200 g) was subjected to hydrodistillation on a Clevenger-type apparatus for 2.5 h according to the method described in the literature [20]. The obtained essential oils were dried over anhydrous sodium sulfate and stored at 4 °C in the dark until they were analyzed.

### Isolation of terpenoids 

Samples from the essential oils of both *T. occidentalis* “globosa” (480 mg) and *T. plicata* (502 mg) were subjected to preparative-TLC (eluent system toluene: ethyl acetate 93:7, silica gel 60 F_254_ pre-coated glass plates, 1 mm thickness). Different zones were obtained after spraying with vanillin reagent and they were extracted with pentane-ethyl acetate 90:10. The extracts were evaporated under reduced pressure (30 °C) down to 2–3 mL and kept in 4 °C until they were analyzed with chromatographic and spectroscopic methods (GC, GC-MS).

### Antimicrobial activity assays

Antimicrobial activity of the essential oils and the isolated terpenoids was determined using the agar dilution technique [21]. For all assays, stock solutions of the tested oils and pure compounds in sterile distilled water with 10% Tween 80 have been prepared at 10 and 1 mg/mL, respectively. Serial dilutions of the stock solutions in broth medium (100 μL of Müller-Hinton broth, Sabouraud broth for fungi and blood agar 10% for oral pathogens) were prepared in a microtiter plate (96 wells). Then 1 μL of the microbial suspension (the inoculum, in sterile distilled water) was added to each well. For each strain, the growth conditions and the sterility of the medium were checked and the plates were incubated at 37 °C and the MICs were determined as the lowest concentrations preventing visible growth. 

### Microorganisms

A panel of microorganisms, including two Gram positive bacteria: *Staphylococcus aureus* (ATCC 25923) and *S. epidermidis* (ATCC 12228); four Gram negative bacteria: *Escherichia coli* (ATCC 25922), *Enterobacter cloacae* (ATCC 13047), *Klebsiella pneumoniae* (ATCC 13883) and *Pseudomonas aeruginosa* (ATCC 227853); as well as three pathogenic fungi: *Candida albicans* (ATCC 10231), *C. tropicalis* (ATCC 13801) and *C. glabrata* (ATCC 28838). Standard antibiotics netilmicin and amphotericin B were used in order to control the tested bacteria and fungi. 

## Figures and Tables

**Table 1:** Quantities (%) of components of the volatiles of four *Thuja* essential oils.

Peak no.	Compounds ^a^	*T.occidentalis* “globosa”	*T.occidentalis* “aurea”	*T. plicata*	*T. plicata “*gracialis”	K.I. ^b^
1.	*α*-Thujene	0.28	0.27	0.31	0.30	930
2.	*α*-Pinene	1.45	1.10	1.26	1.88	939
3.	Camphene	2.44	1.00	0.15	1.52	954
4.	Sabinene	**4.55**	**3.43**	**6.00**	**2.94**	975
5.	Myrcene	-	1.19	1.76	2.08	991
6.	*α*-Terpinene	0.29	0.55	0.69	0.60	1017
7.	*p*-Cymene	-	0.18	0.34	0.28	1025
8.	Limonene	3.17	1.45	1.46	2.26	1029
9.	γ-Terpinene	0.95	1.00	1.28	0.51	1060
10.	Fenchone	4.24	0.17	7.06	2.81	1087
11.	*α*-Thujone (*cis*)	**50.14**	**51.60**	**62.12**	**54.48**	1102
12.	*β*-Thujone (*trans*)	**5.58**	2.70	**7.06**	**6.30**	1114
13.	Camphor	**4.47**	**3.09**	-	2.35	1146
14.	Borneol	0.30	-	-	-	1169
15.	1-Terpineol	-	-	0.34	-	1134
16.	Sabine ketone	-	0.21	0.21	-	1159
17.	Terpinen-4-ol	2.28	**3.28**	**4.66**	**3.11**	1177
18.	*meta*-Methyl-acetophenone	-		0.12	-	1183
19.	*p*-Cymen-8-ol	-	0.25	0.37	0.21	1183
20.	*α*-Terpineol	1.38	0.37	0.20	0.27	1189
21.	Chavicol	-	-	0.19	-	1250
22.	*trans*-Piperitol	0.15	-	-	-	1208
23.	*endo*-Fenchyl acetate	0.99	0.23	-	0.34	1220
24.	Couminal-aldehyde	0.07	-	-	-	1242
25.	Carvone	0.25	-	-	-	1243
26.	Carvacrol methyl ether	-	-	-	-	1245
27.	Piperitone	0.12	-	-	-	1253
28.	Cyclofenchone	0.37	-	-	0.38	1265
29.	(-)-Bornyl acetate	2.48	1.32	0.17	2.63	1289
30.	Sabinyl acetate	-	0.29	0.23	0.42	1291
31.	Carvacrol	0.14	0.23	0.28	0.24	1299
32.	*α*-Terpinelyl-acetate	-	-	0.39	1.72	1349
33.	Geranyl acetate	0.12	0.47	0.45	0.35	1381
34.	*trans*-Cinnamyl acetate	0.08	-	-	0.22	1389
35.	Methyleugenol	-	-	0.17	-	1404
36.	δ-Cadinene	0.10	-	0.16	-	1523
37.	(-)-Caryophyllene oxide	1.35	0.31	-	0.18	1583
38.	β-Oplopenone	-	-	-	0.21	1608
39.	*t*-Muurolol	0.08	-	0.13	-	1646
40.	Rimuene	0.07	**5.61**	1.06	2.83	1896
41.	Beyerene	**8.54**	**11.18**	0.65	**4.55**	1932
42.	(+)-Beyerene-19-ol	-	1.48	0.34	0.59	2221
43.	Kaur-15-en	0.10	-	-	-	2314
44.	*trans*-Totarol	0.26	1.38	0.17	0.18	2314
	**Total (%)**	**96.92**	**94.34**	**94.75**	**96.36**	

^a^ Compounds listed in order of elution from a HP-5 MS column. ^b^Kovats Indices (KI) on HP-5 MS capillary column.

**Table 2:** Quantities (%) of abundant components of different origin *Thuja* leaf essential oils.

Compound	*T. occ globosa*	*T. occ aurea*	*T.occ* [2]	*T.occ* [8]	*T.occ* [11]	*T.occ malonyana* [10]	*T. plicata*	*T. plicata gracialis*	*T. plicata* [6]*	*T. plicata* [6]**	*T. plicata* [7]
*α*-Pinene	1.45	1.10	2.0		1.61	1.9-2.9	1.26	1.88	2.0	1.5	1.47
Camphene	2.44	1.00				0.6-1.2	0.15	1.52	0.4	0.2	
Sabinene	4.55	3.43	5.0		1.76	3.2-7.8	6.00	2.94	6.4	6.3	4.16
Car-4-ene									2.8	2.8	
Limonene	3.17	1.45			1.39	0.8-1.4	1.46	2.26	0.5	0.3	0.80
γ-Terpinene	0.95	1.00			0.25	0.5-1.1	1.28	0.51	0.1	tr	1.17
Fenchone	4.24	0.17	8.0	14.06	12.80	6.7-11.1	7.06	2.81			
*α*-Thujone (*cis*)	50.14	51.60	65.0	49.64	48.73	30.4-40.5	62.12	54.48	76.0	77.5	73.16
*β*-Thujone (*trans*)	5.58	2.70	8.0	8.98	7.85	6.5-9.0	7.06	6.30	7.5	7.8	8.25
Terpinen-4-ol	2.28	3.28			2.50	1.8-3.3	4.66	3.11	1.7	1.8	3.17
*α*-Terpineol	1.38	0.37			0.63		0.20	0.27			0.86
Rimuene	0.07	5.61				1.5-11.4	1.06	2.83			0.30
Beyerene	8.54	11.18					0.65	4.55			0.27

[6]* data for the mature tree; [6]** data for the young tree.

**Table 3:** Antimicrobial activities (MIC mg/mL) of *Thuja* essential oils and their main components.

	Species	*S. aureus*	*S. epidermidis*	*P. aeruginosa*	*E. cloacae*	*K. pneumoniae*	*E. coli*	*C. albicans*	*C. tropicalis*	*C. glabrata*
Essential oils										
*T. occidentalis ‘globosa’*		1.50	1.54	2.15	2.37	2.25	2.75	1.50	1.35	1.48
*T. occidentalis ‘aurea’*		1.12	1.35	1.60	1.95	2.10	2.15	1.75	1.87	1.55
*T. plicata*		0.65	0.50	0.72	0.90	1.25	0.85	1.12	0.95	0.87
*T. plicata ‘gracialis’*		0.75	0.82	0.97	1.24	1.15	0..87	1.45	1.25	1.15
Beyerene		0.90	1.10	1.37	1.25	0.87	1.00	1.50	1.15	1.17
*α*- and *β-*Thujone		0.09	0.10	0.75	0.83	0.65	0.35	0.95	0.77	0.75
Netilmicin		4·10^-3^	4·10^-3^	8.8·10^-3^	8·10^-3^	8·10^-3^	10·10^-3^	-	-	-
Amphotericin B		-	-	-	-	-	-	1·10^-3^	0.5·10^-3^	0.4·10^-3^
